# Single-Session Interventions Embedded Within Tumblr: Acceptability, Feasibility, and Utility Study

**DOI:** 10.2196/39004

**Published:** 2022-07-25

**Authors:** Mallory L Dobias, Robert R Morris, Jessica L Schleider

**Affiliations:** 1 Department of Psychology Stony Brook University Stony Brook, NY United States; 2 Koko San Francisco, CA United States

**Keywords:** web-based intervention, internet intervention, digital intervention, single-session intervention, mental health

## Abstract

**Background:**

Existing mental health treatments are insufficient for addressing mental health needs at scale, particularly for teenagers, who now seek mental health information and support on the web. Single-session interventions (SSIs) may be particularly well suited for dissemination as embedded web-based support options that are easily accessible on popular social platforms.

**Objective:**

We aimed to evaluate the acceptability and effectiveness of three SSIs, each with a duration of 5 to 8 minutes (Project Action Brings Change, Project Stop Adolescent Violence Everywhere, and REFRAME)—embedded as Koko *minicourses* on Tumblr—to improve three key mental health outcomes: hopelessness, self-hate, and the desire to stop self-harm behavior.

**Methods:**

We used quantitative data (ie, star ratings and SSI completion rates) to evaluate acceptability and short-term utility of all 3 SSIs. Paired 2-tailed *t* tests were used to assess changes in hopelessness, self-hate, and the desire to stop future self-harm from before to after the SSI. Where demographic information was available, the analyses were restricted to teenagers (13-19 years). Examples of positive and negative qualitative user feedback (ie, written text responses) were provided for each program.

**Results:**

The SSIs were completed 6179 times between March 2021 and February 2022. All 3 SSIs generated high star ratings (>4 out of 5 stars), with high completion rates (approximately 25%-57%) relative to real-world completion rates among other digital self-help interventions. Paired 2-tailed *t* tests detected significant pre-post reductions in hopelessness for those who completed Project Action Brings Change (*P*<.001, Cohen *d_z_*=−0.81, 95% CI −0.85 to −0.77) and REFRAME (*P*<.001, Cohen *d_z_*=−0.88, 95% CI −0.96 to −0.80). Self-hate significantly decreased (*P*<.001, Cohen *d_z_*=−0.67, 95% CI −0.74 to −0.60), and the desire to stop self-harm significantly increased (*P*<.001, Cohen *d_z_*=0.40, 95% CI 0.33 to 0.47]) from before to after the completion of Project Stop Adolescent Violence Everywhere. The results remained consistent across sensitivity analyses and after correcting for multiple tests. Examples of positive and negative qualitative user feedback point toward future directions for SSI research.

**Conclusions:**

Very brief SSIs, when embedded within popular social platforms, are one promising and acceptable method for providing free, scalable, and potentially helpful mental health support on the web. Considering the unique barriers to mental health treatment access that many teenagers face, this approach may be especially useful for teenagers without access to other mental health supports.

## Introduction

### Limited Accessibility of Existing Mental Health Treatments

Existing mental health treatments have long been inaccessible due to well-established structural (eg, cost, transportation, and time) and individual (eg, stigma and distrust of providers) barriers [1-3]. Furthermore, people experiencing suicidal thoughts or engaging in self-harm often fear the negative consequences of disclosing these experiences (eg, involuntary hospitalization) [4,5], creating additional barriers to treatment among those who may need it most. This is particularly true for teenagers whose caregivers often serve as gatekeepers for their own mental health care [6-8] and who often fear the involvement of a caregiver without their consent [5].

To fill the gap between the *need* for support and *access* to it, many teenagers seek and receive mental health support on the web [9,10]. Specifically, a majority (61%-85%) of teens and young adults have sought information or help for their mental health on the web [10-12]. Teenagers report using web-based mental health resources as a way to obtain free, easily accessible, or anonymous support [12,13], and web-based communities via social platforms (eg, forums and discussion boards) offer a space to share one’s experiences with others who have faced similar challenges [14-17]. Youth facing greater psychological distress are also more likely to search for and discuss topics related to their mental health on the web [15]. Thus, these web-based communities in social platforms represent a unique, underexplored avenue for reaching teenagers in need of mental health support.

### Offering Mental Health Support via Web-Based Social Platforms

Initial efforts suggest that it is *possible* to provide mental health supports that are integrated into existing web-based social platforms. Koko, a nonprofit, web-based mental health platform, provides on-demand peer support [18,19], crisis triage [20], and self-guided interventions on topics such as body image, self-harm, and stress management. People can access Koko services on various messenger apps (eg, Telegram, Facebook Messenger, and Kik) or via direct message channels of certain social networking platforms (eg, Tumblr); in recent studies, a portion of Koko users were directly referred from a social media integration [20]. However, no previous evaluation of Koko’s services has focused entirely on investigating the acceptability and effectiveness of embedding mental health supports within a social networking platform.

### Single-Session Interventions as Embedded Web-Based Support

*Single-session interventions* (SSIs)—brief, targeted interventions designed to be completed within 1 sitting or clinical interaction—provide an especially promising intervention format for reaching young people on the web [21]. Multiple large-scale studies have indicated that SSIs can create meaningful changes for key mental health outcomes. In a recent randomized controlled trial of adolescents (N=2452), a 20- to 30-minute web-based behavioral activation SSI (Project Action Brings Change [ABC]) outperformed a supportive therapy control program at reducing postintervention hopelessness and depressive symptoms 3 months later [22]. Another trial (N=565) found Project Stop Adolescent Violence Everywhere (SAVE), a web-based 30-minute SSI targeting self-injurious thoughts and behaviors in teenagers, improved postintervention self-hatred, and desires to stop future self-harm, relative to an active control [23]. Taken together, substantive evidence suggests the efficacy of web-based SSIs in improving mental health outcomes among young people.

Despite the great *potential* of SSIs to increase access to mental health supports, little large-scale research has evaluated their acceptability and effectiveness when disseminated in web-based settings that young people already routinely access. One study evaluated the acceptability and preliminary utility of a free, open-access platform offering 3 web-based SSIs for youth [24]. After viewing paid advertisements on a social media platform (ie, Instagram), nearly 700 youths clicked on the advertisement and viewed the web-based platform within 6 months. The youths rated all 3 SSIs as acceptable, and analyses indicated significant pre- to postintervention reductions in hopelessness and self-hate among 187 SSI completers. However, to complete an SSI program, the youth had to be (1) aware of the open-access platform and (2) motivated to visit the platform on a separate, unfamiliar website and finish a 30-minute activity. Reducing friction (ie, points of difficulty) between individuals and their technology during key help-seeking thresholds may be particularly important for encouraging greater access to mental health supports on the web [25]. Rather than relying on individuals’ ability and motivation to seek external mental health resources in moments of heightened distress (eg, standalone web-based resources, crisis hotlines, and text lines), SSIs could provide accessible, in-the-moment anonymous supports for young people who are *already embedded* in popular social platforms.

### Considerations for Intervention Design

SSIs designed for these web-based, embedded contexts should be streamlined to improve engagement while retaining their therapeutic value. Once disseminated in the real world, digital interventions often encounter issues with user engagement (ie, low uptake and low completion) [26-28]. Substantially reducing the time and effort required to complete web-based SSIs (eg, condensing content and reducing the number of writing prompts) may improve user engagement, particularly for SSIs delivered as in-the-moment supports at times of elevated distress. Determining which elements of SSIs to condense versus preserve presents a particular design challenge for the creation of SSIs embedded on the web.

Several intervention design principles, drawn from basic research in social psychology, education, and marketing [29], have been theorized to support the use of web-based SSIs for mental health: (1) using neuroscientific evidence to normalize youths’ experiences and boost message credibility; (2) centering youths’ expertise in their own lived experiences; (3) asking youths to share what they have learned with others, using their own words; and (4) providing testimonials from others experiencing similar and relevant challenges [21]. These core principles have been featured in much of the existing research evaluating web-based SSIs [22-24]. However, limited research to date has evaluated whether *very* brief, streamlined SSIs (ie, SSIs much shorter than 30 minutes) that retain these core principles may still provide some benefit as quick, free mental health supports embedded on the web.

### This Study

As a majority of existing mental health treatments remain inaccessible, many young people seek mental health support via social platforms on the web. SSIs are uniquely well positioned for integration within web-based social platforms, providing free, brief, and anonymous mental health support options at scale. However, little research has formally evaluated the acceptability and effectiveness of very brief SSIs designed for this context. This study adapted 3 web-based SSIs (5-8 minutes) that were offered as Koko minicourses on Tumblr, a microblogging and social networking website with 135 million active users monthly [30]. The aims of this study were 2-fold: (1) to evaluate the *acceptability* of SSIs in this context via user data and acceptability ratings and (2) to assess *short-term effectiveness* of all 3 SSIs in improving key mental health outcomes (ie, hopelessness, self-hate, desire to discontinue self-harm) from before to after the SSI. Given the unique barriers to mental health treatment access faced by teenagers, the analyses were restricted to teenagers (ages 13-19 years) where possible. The results may inform continued efforts to increase access to free, anonymous, and timely mental health support options.

## Methods

### Ethical Considerations

For this study, we collected anonymous data exclusively from individuals on Tumblr—all of whom were introduced to the service by either (1) clicking on a featured advertisement from Tumblr (eg, “take control by taking this mood-boosting minicourse”) or (2) direct referral from the platform. As all data were part of a completely anonymous program evaluation, this study was deemed as nonhuman subjects research in consultation with the institutional review board at Stony Brook University. In addition, Koko’s privacy policy and terms of service acknowledge that anonymized data may be shared for research purposes.

### Recruitment and Procedure

The direct referral pathway for each of the 3 SSIs was similar. Users who searched for mental health topics on Tumblr were shown an in-app overlay with links to various resources, such as The National Suicide Prevention Lifeline. A set of over 1300 keywords and their derivations were used to detect terms such as “self-harm” or “depression” as well as slang and obfuscations, such as “sewer-slide” and “'s3lf h@rm.” In addition to links to crisis lines, users were also sent a direct message from Koko through the Tumblr direct message channel. Specifically, they were sent the following automated message from a chatbot called “Kokobot”:

Hi! I’m Kokobot [wave emoji]. I’m working with Tumblr to connect people who are interested in mental health topics. Type “hi” to get started...

Next, users were onboarded to the service and asked to describe a recent negative situation that they have been facing, along with any associated negative thoughts. From there, a set of text-based classifiers for mental health [20,31] categorized the user’s posts and directed them to one of several resources, including peer support, crisis lines, and SSIs. Users could also access any of these services from a main menu at any point while using the platform. Users whose text descriptions were flagged by a crisis model were asked to specify whether their current struggles were related to suicidal thoughts, abuse, eating disorders, or self-harm. If a user disclosed that they were struggling with self-harm, they were shown crisis lines from around the world as well as a link to Project SAVE.

The 3 SSIs were initially introduced as Koko minicourses at 3 separate times: March 2021 (Project ABC), June 2021 (REFRAME), and July 2021 (Project SAVE). Across all 3 SSIs, the data for this study were collected through February 2022. Preintervention data were collected immediately before beginning each SSI (ie, each Koko minicourse), and postintervention data were collected immediately following the completion of a program.

### SSI Programs

#### Project ABC Single-Session Intervention

Project ABC minicourse was a briefer 5- to 8-minute version of the original 20- to 30-minute Project ABC SSI evaluated in earlier randomized trial research [22]. As with the full-length program, the abbreviated Project ABC SSI used principles of behavioral activation, encouraging individuals to “take action” and engage in pleasurable behaviors that align with personal values to boost mood and build self-efficacy. Similar to the original SSI, the brief minicourse involved the following components: (1) psychoeducation, describing how taking values-based *actions* can boost *mood* over time; (2) values assessment, where individuals identify a top value for them (eg, academics, friendships, hobbies, family, or staying active); (3) “action plan” exercise, where individuals develop a personalized plan for engaging in meaningful activities of their choice; (4) roadblock exercise designed to identify real-life obstacles and how to address them; and (5) writing prompts, where individuals can share what they have learned with other Koko users. This shortened version of the Project ABC SSI contained condensed intervention content, fewer writing prompts, and greater emphasis on multiple-choice, interactive options, relative to the original 30-minute program.

#### Project SAVE Single-Session Intervention

Before disseminating as a Koko minicourse, an abbreviated (8-minute) version of the Project SAVE SSI was adapted from an original 30-minute program [32]. Project SAVE was designed to reduce the use of self-harm behaviors to cope with high emotional distress, especially in the context of self-hatred or desires to punish oneself [33]. Similar to the original SSI, the 8-minute version of Project SAVE included evidence-based techniques that are common in cognitive behavior therapies (eg, psychoeducation and secondary distress tolerance skills). Specifically, Project SAVE included four key components: (1) information about how changing *actions* (eg, reducing self-harm behavior, being kinder to yourself) can change *feelings* and *thoughts* for the better; (2) statistics and testimonials from other teenagers with lived experiences of coping with self-hatred or self-harm; (3) alternative coping strategies to use in place of self-harm; and (4) offering advice to others using Koko based on what they learned. Compared with the original, the shortened Project SAVE SSI contained condensed material, fewer writing exercises, and more multiple-choice, interactive options.

#### The REFRAME Single-Session Intervention

The REFRAME SSI (5 minutes) teaches cognitive reappraisal, an emotion regulatory strategy that involves modifying one’s interpretation of stressful situations [34]. A recent multicountry study with 21,644 individuals found that a brief web-based training on cognitive reappraisal reduced negative emotions and increased positive emotions about COVID-19–related stressors [35]. The REFRAME SSI on Koko included similar components, but it did not specifically target COVID-19–related stressors and it did not distinguish between multiple reappraisal strategies (eg, reconstrual vs repurposing). It included (1) a brief introduction to cognitive reappraisal; (2) a simplified description of its neural correlates; (3) practice examples with vignettes taken from the Koko peer support platform; and (4) a set of brief prompts to help users engage positive reappraisals in their own lives (eg, “This could get better because...”; “This isn’*t* 100% my fault because...”). Users were also taught how to practice this skill on the Koko peer support platform and that helping others reappraise may confer positive psychological outcomes [36,37].

### Measures

#### Demographics

Demographic information (age, gender, and race and ethnicity) was collected as part of the preintervention measures for individuals completing Project SAVE.

#### Hopelessness

The Beck Hopelessness Scale-4 is a brief and reliable measure used to assess hopelessness in young people [38,39]. At pre- and postintervention time points for the Project ABC and REFRAME SSIs, responders indicated their agreement “right now, in the present moment” with each of 4 statements (eg, “I feel that my future is hopeless and things will not improve”) on a 4-point Likert scale (1=“absolutely disagree”; 4=“absolutely agree”). Hopelessness scores for each person (4-item average) ranged from 1 to 4, with higher values indicating greater hopelessness. Internal consistency was Cronbach α=.84 and Cronbach α=.89 for pre- and post-Project ABC time points, and α=.81 and Cronbach α=.87 for pre- and post-REFRAME SSI time points, respectively.

#### Self-hate

The Self-Hate Scale is a reliable 7-item measure used to assess self-hatred in young people [24,40]. At pre- and postintervention time points for the Project SAVE SSI, respondents indicated how true each statement (eg, “I hate myself”) is for them, “right now, in the present moment,” on a 7-point Likert scale (1=“not at all true for me”; 4=“somewhat true for me”; 7=“true for me”). The self-hate scores for each person (7-item average) ranged from 1 to 7, with higher values indicating greater self-hate. Internal consistency was Cronbach α=.90 and Cronbach α=.94 for pre- and post-Project SAVE SSI time points, respectively.

#### Desire to Discontinue Self-harm

Desire to discontinue self-harm behavior was indexed using a single item adapted from the Self-Injurious Thoughts and Behaviors Interview–Revised [23,41]. Individuals were asked, “How much do you want to stop purposefully hurting yourself without wanting to die?” and told to rate their answer on a 5-point Likert scale (1=“I don’t want to stop at all”; 5=“I definitely want to stop”). A sixth option (eg, “I have stopped engaging in these behaviors”) was available for individuals not currently engaging in self-harm behaviors. Data for these individuals were not included in the analyses evaluating pre- to post-SSI changes in this outcome.

#### SSI Feedback

After completing all 3 SSIs, users were prompted to provide a quantitative “star rating” of the program, from 1 to 5 stars, where higher star values indicate higher ratings or more positive feedback. In addition, after the intervention, all individuals were asked if they would like to provide qualitative feedback via an optional writing prompt (eg, “Do you have any feedback for us?”).

### Statistical Analysis

#### Power

Previous web-based SSI research suggests small to large within-group effect sizes for key outcomes (including hopelessness and self-hatred) using more naturalistic study designs (ie, not randomized controlled trials) [24]. Thus, we estimated our statistical power to detect a small within-group effect (Cohen *d*=0.20) using a paired 2-tailed *t* test in the smallest of the 3 samples (ie, REFRAME, n=768 pairs).

#### Data Exclusion

We assessed the total number of views, starts, and completions for each SSI, as well as item-level drop-off data within each SSI, to describe broad usage patterns without excluding any data. Demographic and outcome data were only recorded and made available once individuals advanced through to the end of a program and clicked “submit.” All pre-post analyses, star ratings, and qualitative data were therefore conducted and reported within program “completers” (ie, individuals who completed an SSI). In addition, where demographic data were available (Project SAVE SSI data), we restricted our analysis to teenagers (ie, excluding individuals who reported ages outside of 13-19 years). As Project SAVE was designed for teenagers engaging in self-harm, only individuals who self-disclosed recently engaging in self-harm (via the Koko onboarding pathways described earlier in this section) were directed to complete this program.

#### Usage Patterns and SSI Feedback

For each SSI, we evaluated usage patterns and feedback, including the number of people who viewed (ie, opened the first page of the program), started (ie, advanced past the first page), completed (ie, advanced through the entire program and beyond the questions), and provided “star ratings” (ie, quantitative ranking of 1-5 stars, with higher stars reflecting a higher rating) for each SSI. In addition, we calculated the program completion rates (percentage completed out of those who started) and average star ratings for each SSI. To illustrate the types of qualitative feedback each SSI received, we extracted specific examples of positive and negative feedback (see the Results section).

Aggregate-level data were available for views and user dropout, for every page within each SSI, among the entire sample (ie, without focusing solely on program completers). We reported these results by plotting the percentage dropout from the number of views for each page within each SSI.

#### Evaluating Pre-Post Changes

We evaluated pre- to postintervention changes for 4 outcomes: hopelessness (via two separate tests, 1 for Project ABC and 1 for REFRAME) as well as self-hate and desire to discontinue self-harm (Project SAVE only). After checking appropriate assumptions (ie, verifying that pre-post difference scores for each outcome were approximately normally distributed) [42], we performed 2-tailed, paired *t* tests across all outcomes for all programs. We calculated Cohen *d_z_* for paired *t* tests using *t* values, sample sizes, and the Measure of the Effect package in R (version 4.0.0) [43] to evaluate the magnitude of within-group changes for each outcome. For outcomes where difference scores were not normally distributed, we completed an additional sensitivity analysis using the nonparametric Wilcoxon signed-rank test to evaluate within-group changes from before to after the intervention [44]. We applied a false discovery rate correction to all 4 *P* values to reduce the likelihood of false positives [45]. Thus, we interpreted the results for each outcome as significant if the false discovery rate–corrected *P* values were <.05. All analysis code and deidentified data for this study have been made publicly available [46].

## Results

### Sample Characteristics

As Koko provides anonymous mental health support, individuals are not required to provide potentially identifiable demographic information (eg, age, gender, and race and ethnicity) to complete minicourses. Specific demographic information for this study (age, gender, and race and ethnicity) was only available for the Project SAVE SSI, as this minicourse was introduced to Koko *after* Project ABC and REFRAME SSIs had been introduced. For the Project SAVE minicourse, individuals were given the option (but were not required) to report demographic information about themselves.

The Project SAVE analyses excluded individuals who were not teenagers between the ages of 13 and 19 years, resulting in a final sample of 1194 individuals (72.28% of the total Project SAVE data). Among this group, the average age of the individuals who completed Project SAVE was 15.71 (SD 1.83) years. The top three most commonly endorsed gender identities were female (419/1194, 35.09%), nonbinary (189/1194, 15.83%), and not sure (98/1194, 8.21%). The top three most commonly endorsed racial or ethnic identities were White (607/1194, 50.84%); Asian (172/1194, 14.41%); and Hispanic or Latinx (112/1194, 9.38%). Table 1 presents complete details on participant demographics.

**Table 1:** Gender identity and race and ethnicity for Project Stop Adolescent Violence Everywhere (N=1194).

Demographics		Values, n (%)
**Gender**		
	Agender	35 (2.93)
	Androgynous	13 (1.09)
	Female	419 (35.09)
	Female to male transgender	74 (6.20)
	Gender expansive	15 (1.26)
	Gender identity not listed	18 (1.51)
	Gender information missing	219 (18.34)
	Intersex	3 (0.25)
	Male	27 (2.26)
	Male to female transgender	2 (0.17)
	Nonbinary	189 (15.83)
	Not sure	98 (8.21)
	Prefer not to say	26 (2.18)
	Transfeminine gender	2 (0.17)
	Trans man	10 (0.84)
	Transmasculine gender	44 (3.69)
	Transgender	15 (1.26)
	Two-spirited	2 (0.17)
**Race and ethnicity^a^**		
	Asian	172 (14.41)
	Black or African American	66 (5.53)
	Hispanic or Latinx	112 (9.38)
	Native American or Alaska Native	23 (1.93)
	Native Hawaiian or other Pacific Islander	12 (1.01)
	White	607 (50.84)
	Prefer not to answer	125 (10.47)

^a^Individuals could select multiple racial and ethnic identities.

### Statistical Analysis

#### Power

Power analyses indicated that we had 99% power to detect a small effect size (Cohen *d*=0.20) using a 2-tailed paired *t* test in the smallest of the 3 current samples (REFRAME, n=768 pairs).

#### Usage Patterns and SSI Feedback

##### Project ABC

**Figure 1:**
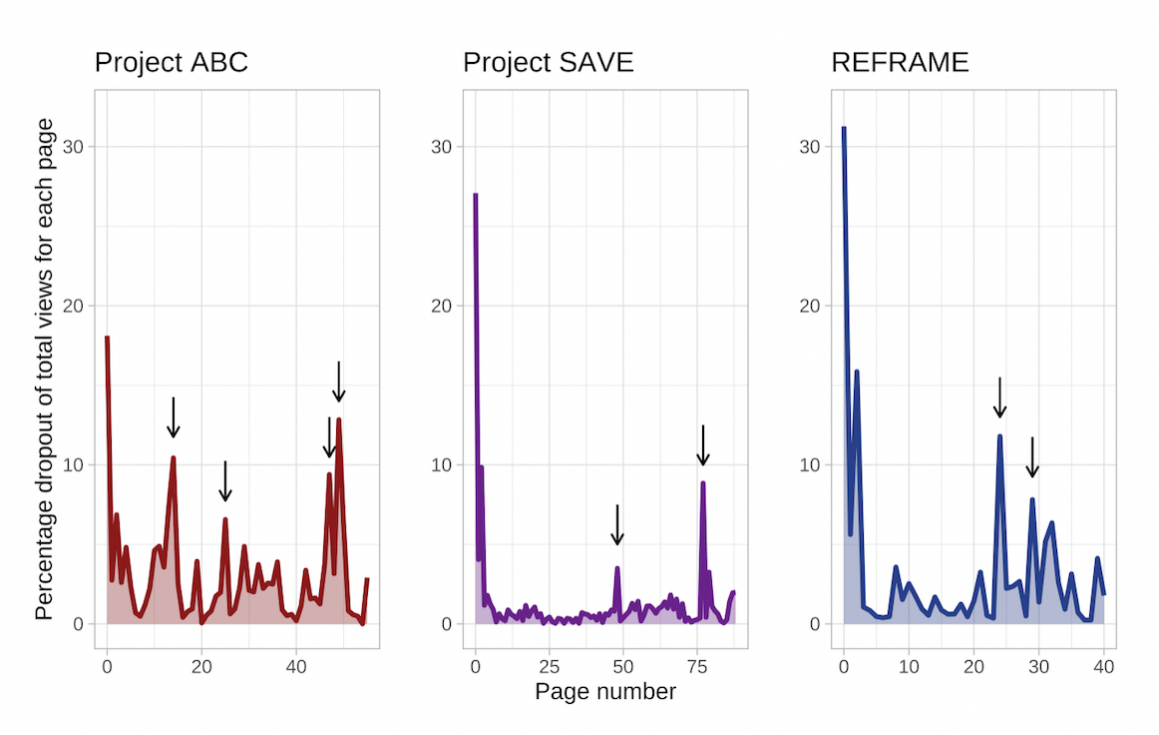
Percentage dropout on each page of all 3 single-session interventions (SSIs), out of the number of individuals who viewed that page. Arrows reflect points where writing prompts were introduced, in each of the 3 SSIs. Spikes in dropout tended to occur after initially opening each SSI, as well as on pages requesting written responses. ABC: Action Brings Change; REFRAME; SAVE: Stop Adolescent Violence Everywhere.

Project ABC was viewed 17,620 times, started 14,434 times, and completed 3679 times—with a 25.49% (3679/14,434) completion rate among starters across the 12-month study period. Among the Project ABC completers, 3412 (92.74%) provided a star rating with an average star rating of 4.27 (SD 0.94; median 5) stars. In total, 1217 (33.08%) provided qualitative feedback on Project ABC (see Table 2 for examples of user feedback). The dropout percent of the number of views for each page for all 3 SSIs is plotted in Figure 1. Notably, high levels of dropout (relative to page views, 7%-13%) consistently occurred on pages where Project ABC users were asked to respond to a writing prompt.

Finally, Project ABC received far more views, starts, and completions than either of the other two SSI programs (4065 and 2174 views for Project SAVE and REFRAME, respectively), as Tumblr advertised the Project ABC SSI as a featured minicourse between December 2021 and February 2022, resulting in higher traffic to this SSI.

**Table 2:** Examples of positive and critical feedback for all single-session interventions (SSIs).

SSI	Positive feedback	Critical feedback
ABC^a^	“Thanks y’all! been dealing w some serious mental health issues and having places to remind me of my agency and joy is really helpful.”	“Despite the great intentions and work I think there is situations that are very complicated and having this intermediate bot, very few info of the person you are helping it’s too simplistic.”
	“this was really really helpful and i’m seriously going to try my goal/plan. I also feel awake and motivated enough to study. I’d love to see more in the future.”	“This helped me see get through my rain cloud but now I kinda feel stressed.”
	“Hey, this was surprisingly well done. I went in expecting it would be terrible. But you’re making mental health a really approachable topic for people. Thanks for working on reaching out to others. I’d love to see you continue with these mini-courses.”	“This is a good idea, but only works for the things one has control over. If you are terminally ill, just lost a loved one or in another uncontrollable challenge, none of these can help.”
REFRAME^b^	“This is such a great way to de-stress, I mean re-frame your stress, it’s definitely a bit helpful.”	“Less simplification”
	“I decided to pick up my phone and do this while I was procrastinating. It’s so crazy how this seemingly small task changed my perspective.”	“i think that one of the problems i see is that this requires people to be more specific and there isn’t a sense of connection.”
	“I love this so much!! It actually made me feel better, which I didn’t think it would! Thank you <3.”	“Please include physical stress reducers, as well. It is difficult to focus on reducing stress when I can’t properly string thoughts together.”
SAVE^c^	“This is amazing. My thoughts of self-harm faded a bit, and prompted me to do the things alternative to when I have self-hate thoughts.”	“It would’ve been more helpful if reasons for self-harm outside of self-hatred were explored. I’m currently dealing with external circumstances that are overwhelming me and my response is to harm myself. It feels like the only way to release my emotions.”
	“This is the most convincing thing I’ve ever heard as to why not to self harm. Thank you so much this is so helpful.”	“I have dealed with this so long that there wasn’t really anything new to me, so it really didn’t help.”
	“Thank you. This course has definitely calmed me down when I was having a breakdown and thinking of hurting myself.”	“Talk about slowly building up to recovery instead of just jumping right in. Talk about how to deal with intense emotions and more.”

^a^Project Action Brings Change (ABC) SSI.

^b^REFRAME SSI.

^c^Project Stop Adolescent Violence Everywhere (SAVE) SSI.

##### Project SAVE

In 12 months, Project SAVE was viewed 4065 times, started 2961 times, and completed 1652 times; 55.79% (1652/2961) of those who started Project SAVE completed it. After excluding individuals with ages outside our desired range (13-19 years), 1194 observations remained for analysis. Among those who completed the minicourse, 954 (79.90%) provided a star rating for Project SAVE, with an average rating of 4.22 (SD 0.97; median 5) stars. A total of 209 (17.50%) participants provided qualitative feedback on Project SAVE (Table 2). Similar to the Project ABC SSI, Project SAVE observed higher dropout rates (relative to page views, 3%-9%) on pages where users were asked to enter a written response (Figure 1).

##### REFRAME

REFRAME was viewed 2174 times, started 1498 times, and completed 848 times within 12 months (848/1498, 56.60% completion rate among the starters). Among REFRAME completers, 732 (86.32%) provided a star rating for the REFRAME SSI, with an average rating of 4.31 (SD 0.93; median 5) stars; 246 (29.01%) provided qualitative feedback on the REFRAME minicourse (Table 2). Consistent with dropout patterns for the other 2 SSIs, REFRAME observed higher dropout rates (relative to page views, 8%-12%) on pages requesting writing from users.

#### Evaluating Pre-Post Changes

Among individuals who completed Project ABC, hopelessness significantly decreased from before to after the SSI (*t*_3,565_=−48.48, *P*<.001). Similarly, individuals who completed the REFRAME SSI reported significant reductions in hopelessness from before to after the intervention (*t*_767_=−24.41, *P*<.001). Project SAVE completers reported significant pre- to postintervention reductions in self-hate (*t*_1,007_=−21.30, *P*<.001) and an increase in desire to discontinue self-harm (*t*_839_=11.48, *P*<.001). All the means, SDs, and within-group effect sizes are listed in Table 3.

**Table 3:** Means, SDs, and effect sizes for all single-session intervention (SSI) outcomes.

Outcome and SSI		Before the SSI, mean (SD)	After the SSI, mean (SD)	Cohen *d*_z_ (95% CI)
**Hopelessness**				
	ABC^a^	2.60 (0.78)	2.16 (0.80)	−0.81 (−0.85 to −0.77)
	REFRAME^b^	2.86 (0.74)	2.31 (0.78)	−0.88 (−0.96 to −0.80)
**Self-hate**				
	SAVE^c^	5.69 (1.27)	5.07 (1.64)	−0.67 (−0.74 to −0.60)
**Desire to stop harm^d^**				
	SAVE	2.63 (1.20)	2.97 (1.32)	0.40 (0.33 to 0.47)

^a^Project Action Brings Change (ABC) SSI.

^b^REFRAME SSI.

^c^Project Stop Adolescent Violence Everywhere (SAVE) SSI.

^d^Desire to discontinue self-harm behavior.

**Figure 2:**
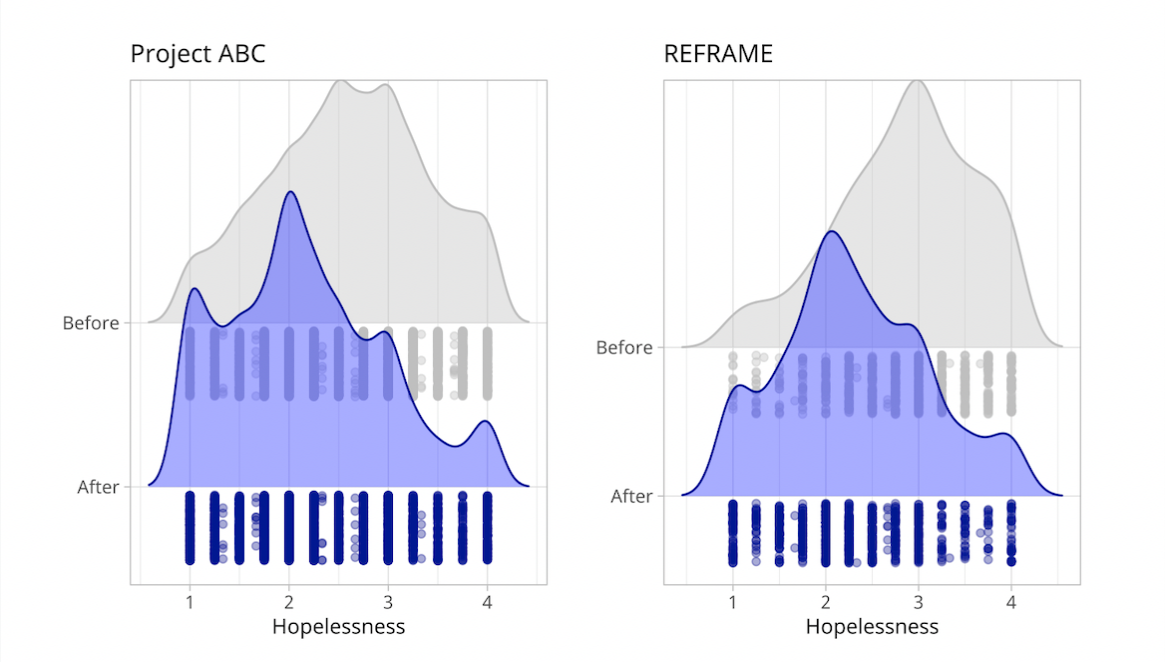
Hopelessness ratings before and after the Project Action Brings Change (ABC) single-session intervention (SSI; left) and before and after the REFRAME SSI (right). Higher scores reflect higher levels of hopelessness.

**Figure 3:**
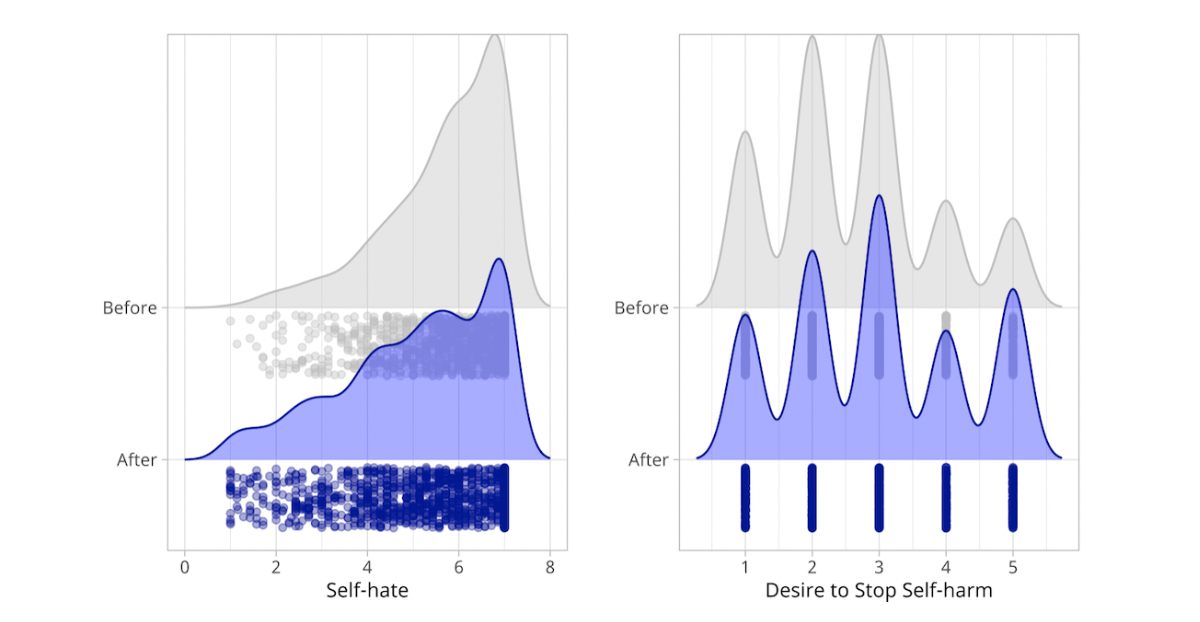
Self-hate ratings before and after the Project Stop Adolescent Violence Everywhere single-session intervention (left), where higher scores reflect higher levels of self-hatred. (Right) Desire to stop future self-harm behavior, where higher scores reflect higher desire to stop future self-harm.

In addition, Wilcoxon signed-rank tests were performed as sensitivity analyses for all outcomes due to the relatively nonnormal distributions of difference scores. For all outcomes, results were consistent with *t* test analyses, indicating significant pre-post reductions in hopelessness (*P*<.001; Project ABC and REFRAME) and self-hate (*P*<.001; Project SAVE), as well as a significant increase in desire to discontinue self-harm (*P*<.001; Project SAVE) from before to after the intervention. Distributions for all outcomes at pre- and post-SSI time points are shown in Figures 2 and 3.

## Discussion

### Principal Findings

Within 12 months, 3 very brief (5-8 minutes) web-based SSIs were viewed >18,800 times and completed >6100 times. Offered as Koko minicourses and embedded within a popular social platform, all 3 SSIs received high-quality ratings (average ratings >4 out of 5 stars). Of the participants who started an SSI, between 25% and 57% of them completed one. Among those who completed Project ABC and REFRAME minicourses, individuals reported a decrease in hopelessness from before to after the SSI. For those who completed Project SAVE, individuals reported decreased self-hatred and increased desire to stop future self-harm behavior from before to after the intervention. This study represents a real-world evaluation of the acceptability and short-term utility of web-based SSIs as very brief, anonymous, “in-the-moment” mental health supports that can be integrated within major social platforms such as Tumblr.

### Comparison With Prior Work

Consistent with existing research on web-based SSIs [24], all 3 interventions were (1) rated as acceptable by users and (2) associated with improvements across primary outcomes. Furthermore, despite reducing intervention length by *nearly 75%*, effect sizes for these 5- to 8-minute SSIs were remarkably similar to effect sizes for SSIs designed to last 30 minutes (Cohen *d_z_* =0.71 vs 0.81 (hopelessness) and Cohen *d_z_* =0.61 vs 0.67 (self-hate) for full and abbreviated SSIs, respectively) [24]. These results support a growing body of evidence that suggests even extremely brief digital interventions may improve clinically relevant outcomes [20,47].

One possible reason for the relative similarity of postintervention effect sizes observed in this study (5-8–minute SSIs) versus within-group effects in earlier randomized trials (30-minute SSIs) [22,23] might be the shared design features and principles across the “brief” and “very brief” versions of these interventions. Given that these core principles have been featured in all versions of these SSIs to date, it is possible that these common principles are more important than the exact length of the SSI. Future research should formally evaluate this possibility in a head-to-head trial by comparing SSIs of various lengths.

Our results may also indicate the *real-world utility* of these very brief web-based SSIs. Naturalistic study designs similar to that used in this study are crucial for evaluating acceptability and utility among web-based SSIs, as randomized trials overestimate user engagement levels for unguided, digital mental health interventions—often producing usage rates that are *4 times* greater than rates observed in real-world settings [27]. Once these interventions are disseminated outside of randomized trials, a small minority of individuals (0.5%-28.6%) complete all content [28]. Therefore, relative to real-world completion rates of other digital self-help interventions for mental health, completion rates for the 3 SSIs tested in this study (25%-57%) were extraordinarily high.

Low real-world completion rates for self-help digital interventions, especially relative to guided digital supports [26,48,49], are unsurprising; unguided supports require substantial emotional and mental bandwidth (eg, energy, sustained attention, and motivation) to find and engage with an unguided mental health support tool at the very moments when these capacities may be at their lowest (eg, when experiencing elevated distress). Our study sought to minimize the bandwidth necessary to engage with 3 single-session, unguided supports by (1) dramatically reducing their length and (2) bringing these interventions to web-based spaces where people already are. Future research should explicitly assess whether these strategies improve completion rates across other unguided digital mental health supports.

Embedding SSIs within a popular social platform (Tumblr) also likely increased the *visibility* and uptake of these SSIs. For example, after 6 months of recruitment using paid advertising and leveraging substantial media coverage, 1 study found that 700 youths viewed an open-access, web-based mental health platform featuring 3 SSIs [24]. The 3 SSIs in this study received a combined ≥18,800 views within 12 months—nearly 6240 of which were views of interventions that were never featured in a Tumblr-based advertisement (Project SAVE and REFRAME). These numbers suggest considerable interest in accessible, anonymous, “in-the-moment” support options, as well as the potential to *sustainably* offer these supports at high scale and relatively low cost.

Notably, existing research identifies safety as a primary ethical concern for researchers and stakeholders interested in using digital mental health tools among youth [50]; many digital resources lack evidence or clinical validation and others may not provide sufficient protection for sensitive user data. By using SSIs that have previously been evaluated in rigorous randomized trial research [22,23] and by collecting anonymous pre- and post-SSI user data, in this study, we aimed to mitigate these common concerns. Thus, calls for clearer guidelines for safety and quality in mental health apps [51] should also be accompanied by clearer guidelines, both for academic- and industry-based program developers and researchers, on how to responsibly and ethically disseminate mental health resources via partnerships with social networking platforms.

### Strengths and Limitations

This study has several strengths. Although randomized trial research overestimates user engagement for digital interventions [27], it constitutes much of the existing research on web-based SSIs to date [22,23,52]. Therefore, the naturalistic design of this study provides a valuable, more accurate estimate of the potential for web-based SSIs to offer mental health supports at scale. Koko’s partnership with Tumblr also made it possible to offer anonymous, “in-the-moment” support for people seeking mental health–related content within the platform, rather than requiring individuals to search for external resources. Finally, we conducted a fine-grained analysis of usage data (ie, dropout) going item by item for each SSI. It was especially valuable to recognize high levels of user dropout relative to page views (3%-13%) on pages requesting written responses. Further studies should seek to understand patterns in user dropout and how they may inform digital intervention design. In addition, future evaluations may also want to expand upon this study by conducting smaller-scale usability testing [53] and research explicitly designed for the in-depth analysis of rich qualitative user feedback [54].

In addition to the aforementioned strengths, this study has some limitations. First, demographic information was not available for all individuals included in this study. Demographic information that *was* available from 1 of the 3 SSIs indicated substantial missing data (several users reported skipping demographic items to ensure they could not be identified). Black, Hispanic, and Asian youths consistently access mental health treatment at lower rates than their non-Hispanic White peers [55,56], yet up to 90% of youths in mental health clinical trials are White [57,58]. Digital interventions provide one possible avenue toward reducing disparities in access to mental health resources among racial and ethnic minority populations [59,60]; a vast majority of adolescents in the United States have internet access via either smartphones or desktops or laptops (88% and 95%, respectively) [61]. While there are disparities in broadband access by socioeconomic status, geographic region, educational attainment, and race and ethnicity, some evidence suggests that these gaps in access may be decreasing [62]. Should SSIs hope to provide truly *accessible* and *equitable* mental health supports, they must be accompanied with consistent evaluations of who has access to them, with researchers actively working to prevent further (or exacerbated) inequity via a new treatment modality [60].

In addition, as is often the case in web-based mental health support research [24,63,64], cisgender boys were underrepresented in this sample. Given unique barriers to seeking mental health treatments faced by cisgender boys and young men (eg, internalized masculine gender norms, such as “toughness”; elevated mental health stigma) [65,66], future mixed methods research may want to evaluate whether boys view SSIs as a less stigmatizing or more approachable mental health support option.

Finally, given that this study represents an unmasked evaluation of within-group intervention effects in a completers-only sample, our ability to draw causal inferences was limited. For example, those who completed an SSI may have provided more positive star ratings for the programs than those who exited before finishing the program. However, 2 of the 3 SSIs featured in this study have demonstrated positive effects on identical outcomes in large-scale, triple-masked, and randomized research [22,23]. SSIs have shown considerable promise in multiple studies and study designs.

### Conclusions

Very brief SSIs (5-8 minutes) can be embedded within web-based social platforms as anonymous, in-the-moment mental health supports capable of reaching many individuals within months. Among those who complete these SSIs, individuals generally rate them as acceptable, and pre-post evaluations suggest that they may be helpful in reducing hopelessness and self-hate as well as in increasing the desire to stop self-harm. These pre-post findings, combined with results of earlier randomized trial research, suggest that SSIs delivered in this context may be a sustainable approach for providing the much-needed mental health resources, particularly for teenagers who may not have access to other mental health supports. Considering the substantial unmet need for mental health care among teenagers in the United States [67], SSIs may provide a valuable and complementary source of mental health support among a broader ecosystem of mental health treatment options (eg, school- and community-based programs, mental health screening, and gatekeeper training), all of which are necessary for reducing mental illness at scale.

## Abbreviations

ABC: Action Brings Change
SAVE: Stop Adolescent Violence Everywhere
SSI: single-session intervention
